# A qualitative study to understand drivers of psychoactive substance use among Nepalese youth

**DOI:** 10.1371/journal.pone.0259021

**Published:** 2021-11-05

**Authors:** Tulsi Ram Bhandari, Bhushan Khatiwada, Bibika Rajbhandari, Amy Bestman, Sabuj Kanti Mistry, Binod Rayamajhee, Lal B. Rawal, Uday Narayan Yadav

**Affiliations:** 1 Department of Public Health, School of Health and Allied Sciences, Pokhara University, Pokhara, Nepal; 2 Torrens University, Pyrmont Campus, Sydney, Australia; 3 Centre for Research Policy and Implementation, Biratnagar, Nepal; 4 The George Institute for Global Health, University of New South Wales, Sydney, Australia; 5 Centre for Primary Health Care and Equity, University of New South Wales, Sydney, Australia; 6 School of Optometry and Vision Science, Faculty of Medicine and Health, UNSW, Sydney, Australia; 7 Department of Infection and Immunology, Kathmandu Research Institute for Biological Sciences, Lalitpur, Nepal; 8 School of Health Medical and Applied Sciences, Central Queensland University, Sydney, Australia; 9 National Centre for Epidemiology and Population Health, The Australian National University, Canberra, Australia; Global Center for Research and Development (GCRD), UNITED STATES

## Abstract

**Background:**

Psychoactive substance use among youth is an emerging public health issue in Nepal. This exploratory study aimed to better understand the drivers of psychoactive substance use among Nepalese youth in Rupandehi district of Nepal.

**Materials and methods:**

This study used a qualitative approach for data collection. Both in-depth interviews (IDI, seven participants) and focus group discussions (FGD, 13 participants) were conducted among study participants who self-reported as psychoactive substance users or had history of psychoactive substance use. Participants for IDI were aged between 11 and 24 years and between 18 and 35 years old for FGDs. Semi-structured interview guides were prepared separately for IDIs and FDGs. Interviews were conducted in Nepali language and were audio recorded, which were there transcribed and translated into English for coding and analyses. In addition, interviews notes were taken by two research assistants. An inductive thematic analysis was used to analyze the data.

**Results:**

This study identified a range of drivers of psychoactive substances use among Nepalese youths. Themes included (i) socio-cultural factors, (ii) individual factors, (iii) academic environment, (iv) physical environment and the (v) influence of media. The socio-cultural factors were categorized into sub-themes of family relationships, ethnic identity and psychoactive substance use and lack of social acceptance. Individual factors included peer pressure, stress relief and coping with financial challenges. Accessibility and availability of psychoactive substances in the surrounding environment and lack of monitoring and reinforcement of rules/ law and regulations were other drivers to psychoactive substance use among this Nepalese youth cohort.

**Conclusion:**

Our study identified several important drivers of psychoactive substance use among youth in the Rupandehi district of Nepal. Future works are anticipated to further explore youth initiation and use of psychoactive substances and support the design of interventions that address these risk factors to reduce and prevent subsequent harms.

## Background

Psychoactive substances include alcohol, tobacco, licit and illicit drugs, that when consumed, can harm the mental wellbeing of an individual [1]. In 2017, the United Nations Office on Drugs and Crime (UNODC) estimated 271 million people of age group 15–64 years used any form of drugs and 35 million people suffered from disorders associated with psychoactive substance use [2]. By the end of 2017, 11.8 million deaths were recorded globally due to direct and indirect use of psychoactive substances (predominantly tobacco, alcohol and drugs), out of which 11.4 million were premature deaths [3]. According to the World Health Organization (WHO) Global Health Estimates, in 2019 the proportion of Disability Adjusted Life Years (DALYs) due to alcohol use disorders globally was 0.76% and 0.78% due to drug use disorders [4] whereas, in Nepal, DALYs were 0.5% and 0.29% due to alcohol and drug use disorders, respectively [5, 6].

In Nepal, sale of tobacco and alcohol products by non-licensed vendors have been restricted by law [7, 8]. For example, it is illegal to sell tobacco products to individuals younger than 18 years [7] or alcohol to individual under 21 years [8]. However, despite these laws, it has been estimated that over 21,000 children between 10–14 years consume tobacco each year in Nepal [9]. Additionally, the narcotics control act of Nepal does not allow any person to consume cannabis/marijuana, with penalties such as one month behind the bars or a fine of NRs. 2000 (17 USD) for possession, however these vary on the amount of cannabis/marijuana possessed by an individual [10]. The most prevalent psychoactive substances used in Nepal are cannabis, tranquilizers, and opiates and the highest number of users has been reported from Bagmati Province, capital city of the country [11]. The influence of psychoactive substance misuse during early adolescence has been linked to entertainment, relaxation, curiosity, peer, and media influence [12].

The Government of Nepal survey conducted in 2019, found 130,424 people were using drugs in Nepal with 5.06% average annual growth from 2013 to 2019. Males accounted 93.3% of drug users (females account 6.7% of users) and 76.2% were below of age group 30 years [11]. According to another study conducted in Nepal in 2018 with 387 psychoactive substance users, 10.8% of psychoactive substance users began at 13–15 years and 44.4% started using psychoactive substances between 16 and 20 years [13]. Psychoactive substance use among youths is an important issue that effects both physical and mental health outcomes of youths [14]. Early-onset use of psychoactive substances has been linked to a range of health and social issues including poor physical and mental health, inadequate school performance, unemployment, substance use disorder, and seclusion, a strained relationship with family [15]. Previous research has also identified that the initiation of psychoactive substance use from early adolescence, can lead to cumulative public health challenges [16]. Evidence suggests that the high prevalence of adolescents using psychoactive substances in Nepal is significantly associated with parental use and cultural acceptance of psychoactive substances [17, 18]. However, to date, there is scarce qualitative research that enables deeper understanding of experiences, phenomena and context of psychoactive substances use in Nepal, especially among youths. Thus, the current study was conducted to understand drivers of psychoactive substance use among Nepalese youth in Rupendehi district of Nepal.

## Materials and methods

### Research design

This qualitative study used an interpretivist epistemological view to understand the drivers of psychoactive substance use among Nepalese youth in the Rupandehi district of Nepal [19]. This study used both in-depth interviews (IDI) and focus group discussions (FGD) in order to capture holistic description of the phenomenon. IDIs were selected to allow participants to share experiences openly in a safe environment without hesitation and allows researchers to pursue new themes that emerge during the interview. FDGs were chosen to explore diverse views on a particular phenomenon through group discussion and interaction of prominent issues [20].

### Participant’s recruitment and sampling

Participants for this study were recruited from rehabilitation and treatment centers of Rupandehi district. Purposive sampling was used to recruit participants to ensure sampling variation across age, ethnicity, education status, occupation and economic status of the adolescents. Participants representing various ethnic groups were included; upper caste (Brahmin and Chhetri), indigenous groups (Newar, Tamang) and marginalized communities (Dalit). We only included participants who had reported previous use of psychoactive substances and excluded individuals diagnosed with severe psychiatric disorders, intent to harm self and others, or failed to produce assent consent.

### Data collection

Data were collected from August 2017 to April 2018. This study recruited seven participants for face-to-face IDIs aged between 11 and 24 years. Each IDI lasted 18–25 minutes. Thirteen participants of aged 18–35 years were recruited for FGDs. Two FGDs were conducted at two different settings (one with a group of six participants and another with seven). Duration of FGDs were 40–45 minutes. All FGDs and IDIs were conducted in Nepali language by one of the investigators where notes were taken by two research assistants and were audio-recorded. Interviews were conducted in a comfortable environment to ensure open discussion and encourage all participants to describe their experiences. Questions and sub-questions invited open-responses and related to attitudes and perceptions of adolescents and reasons behind the early initiation of psychoactive substances. The scripts of data were read, re-read and discussed between two investigators to decide the point of saturation. After reaching saturation point, data collection process was topped.

### Trustworthiness of the study

Validity and reliability of the findings were measured in terms of trustworthiness which comprises credibility, transferability, dependability and conformability of the study participants [21]. The investigator who conducted the IDIs and FGDs had a public health background and shared the same language and culture with majority of the study participants. This supported investigators to build rapport and ask appropriate follow up questions. Participants were genuinely willing to take part in the study and provided relevant information freely. The collected data were transcribed, translated, coded and analyzed by four authors and were checked by two lead authors (TB and UNY) for accuracy and consistency of the translations. Analyzed results were also checked by lead authors to examine any discrepancies and data obtained through both FGDs and IDIs were triangulated for similarities and variation [22].

### Ethics approval

This study protocol was reviewed and approved by the Nepal Health Research Council Ethics committee, and approval from the District Education Office, Rupandehi, was also obtained prior to commencing field data collection. Written assent consents were obtained from parents of participants who were below 18 years old. Written informed consents were obtained from the rehabilitation centers and study participants. The confidentiality of all the participants and information obtained was maintained.

### Data analysis

Data were analyzed using an inductive thematic approach, as suggested by Braun and Clarke [23]. Initial codes were developed, and researchers met to discuss the validity of codes in a broader context. The investigators reviewed the transcripts verbatim then developed codes and higher order themes until consensus was reached. The codes were critically analyzed to develop sub-themes and themes. Given that qualitative data was translated into English, some quotes have been edited to increase clarity for readers. Where quotes have been changed, these have included minor edits to language, however the meaning of quotes has remained same.

## Results

Out of 20 participants, 18 were male and two were female and half (n = 10) of participants did not reveal their ethnic identity. Among ten participants who provided information on ethnic identity, five were from indigenous groups, 2 from Dalit (so called low caste/ untouchable caste according to traditional Hindu caste system) and two from so called higher caste (one from Chhetri and another from Brahmin). There were total of 4 participants who were below 18 years old.

Five broad themes socio-cultural factors, individual factors, physical environment, academic environment and influence of media emerged from the data analysis of both FGD and IDI. These have been presented in **Table 1** and have been explained below.

**Table 1:** Themes and sub-themes on drivers of psychoactive substance use among Nepalese youth.

Themes	Sub-themes
A. Socio-cultural Factors	• Family relationships
	• Ethnic identity and psychoactive substance use
	• Lack of social acceptance
B. Individual factors	• Peer pressure
	• Source of stress relief
	• Financial challenges
C. Physical environment	• Accessibility and availability of psychoactive substances in the surrounding environment
	• Monitoring and enforcement
D. Academic environment	
E. Influence of media	

### A. Socio-cultural factors

Socio-cultural factors included active engagement of people and, the cultural practices and influence of people, which impacted psychoactive substance use. This included environments created by families, cultural identity, cultural differences, attitudes and behaviors of people, parenting practices, ethnic identity and societal discrimination. Based on the information provided by participants, we identified the following sub-themes, youth and family relationships, role of peers, ethnicity and social acceptance.

#### i) Family relationships

Participants described lack of supervision such as poor parental care, less support and minimal attention to youth as reasons for initiation and engagement in psychoactive substance use. Some participants referred to the impact of parental separation on their mental health and the role of psychoactive substance use in coping with resulting increased mental stress. For example, one participant from the Dalit community said:

*“I got involved* [with psychoactive substances] *when I was seven years old while studying in a government school*. *My dad divorced my mum*. *You can imagine how much stress I had*, *and I could not bear that stress*. *To cope with that*, *I started using psychoactive substances”* [Male participant, 12 years, Dalit community, IDI]

Some participants described that detachment from family members or the absence of family members to guide during adolescence period forced them to engage in psychoactive substance use. These participants mentioned that they used psychoactive substances as stressor and loneliness solace. One participant from the FGD explained after his parents separated, his mother went overseas to earn money. While he was without his mother, his elder brothers from the community who were involved in psychoactive substance encouraged him to initiate psychoactive substance use:

*“After my mother left when I was eight years old there was no one to spend time with me at home*. *So*, *I started spending time outside and my cousins involved me in using psychoactive substance (drugs like Brown sugar*.*”* [Male Participant, 14 years, FGD].

Lack of attention from parents was also identified as a factor that influenced youth psychoactive substance use. Participants noted family members poor knowledge of youth activities, lack of parental affection, and lack of involvement of parents in their adolescent daily life. Some of the participants from the FGDs expressed parental inability to understand how the complexity of youth problems (for example, low grades in school, financial problems, stigma based on caste or ethnicity) led youths to escape through psychoactive substance use. One participant of FGDs told investigators that his parents adopted very cruel strategies in response to their psychoactive substance use.

*“My family failed to understand me when I involved into drugs; they gave me different tortures in a sick mood (electric shock*, *canning*, *restricted me to go out*, *etc*.*)*. *This forced me to engage more deeply into drugs use*.*”* [Male Participant, 19 years, Indigenous, FGD].

Some of the participants in IDI mentioned that poor parental monitoring and limited concern around adolescents’ activities prompted adolescents to become involved in psychoactive substance use, such as smoking cigarette and progression into the use of ‘hard’ psychoactive substances (drugs, nitrogen tablets, injection). One participant said,

*“I think family members were responsible as well because they never asked for reasons behind asking the money to me*. *They (parents) usually provided me large sums of money*, *which is the main reason for getting indulged in these behaviors (using drugs)*.*”* [Male Participant, 24 years, FGD]

*“Family and friends are responsible for an equal amount*. *Nowadays*, *family members are more focused on earning money and in a way*, *they don’t care about adolescents*. *This leads to the development of the circle (people using* psychoactive substance*)*.*”* [Male participant, 23 years, Chhetri, FGD].

#### ii) Ethnic identity and psychoactive substance use

In Nepal, some ethnic groups such as Dalits, Indigenous groups like *Rai*, *Magar*, *Newar*, *Sherpa* and *Limbu* have a traditional value where they make alcohol in their own home to offer God and drink alcohol in festivals like Dashain or any family occasions like marriage or death ceremonies. Participants from indigenous groups identified that psychoactive substance use, mainly alcohol and tobacco use, as normal behavior. Nearly half of participants (n = 8) explained these cultural links as influential factor to their use of psychoactive substances:

*“My mother used to make alcohol at home*, *and we* [siblings] *used to sell it*. *She* [mother] *used to ask me to taste alcohol to find the strength of alcohol and later my body asked for a higher alcohol dose*, *and then I became an alcohol addict*.*”* [Male participant, 24 years, Indigenous, FGD].

#### iii) Lack of social acceptance

The majority of the participants in IDI mentioned that their history of psychoactive substance use mean they were not accepted in society, even after they got treatment for addiction. These attitudes pushed some to relapse into addictive habits:

*“After leaving the rehabilitation center it was difficult for me to adjust to society*. *They call names like*: *‘jadhya’ (alcoholic) and ‘dhule’ (drug addict)*. *Even if I am trying to do something good*, *the family and society don’t trust me*, *which forced me get use drugs again*.*”* [Male participant, 30 years, FGD].

*“If my friends and families had supported at that time (after returning from rehabilitation)*, *you [*researcher*] would not see me here [*rehabilitation center*] today again*.*”* [Male Participant, 34 years, FGD].

One of the IDI participants mentioned that she was maltreated (emotional abuse) by the family members which forced her to use psychoactive substances again after returning from rehabilitation.

*“My husband has a foreign ID (as my father-in-law is a foreign soldier) and he left for a foreign country to strengthen our financial status*. *After he left*, *the family members of the husband-side started giving torture [verbal and physical abuse] to me in a different way*, *and this is why I get into drugs use again (brown sugar*, *nitrogen tablets)*.*”* [Female participant, Indigenous community, 22 years, IDI].

Few participants from FGD expressed that psychoactive substance users should be given a chance in society to work or share their stories which would dissuade other young Nepalese people to use psychoactive substances.

*“People with history of drug use should get income generating opportunities in the community*. *But society and family do not trust drug users*. *They [*psychoactive substances users*] are always blamed for any wrong things if happen in community such as if someone stole anything*, *the community people first reach to drug users to check if they stole it*. *This forces us* [users] *to return to drugs”* [Male participant, 28 years, FGD].

### B. Individual factors

Many factors that operate at individual level impedes decision making process among youths, resulting their engagement in psychoactive substance use. Peer pressure, source of relief and financial challenges are sub-themes that explain individual factors related to psychoactive substance use.

#### i) Peer pressure

Most participants from both FGD and IDI believed that friends and peer groups were a key factor in initial psychoactive substance use (particularly Marijuana, Cannabis and alcohol use). This included direct or indirect influence or pressure from friend circles for psychoactive substance use.

*“I started with "Chang" [a local alcoholic drink] at the age of 15 during my school life*, *as I was part of the bad circle [psychoactive substance users] in my school*. *Later on*, *I became resistant to change and decided to use other drug use such as syringes*, *brown sugar*.*”* [Female participant, 22 years, IDI].

The majority of participants from the FGD and IDI (all age groups and genders) shared their experiences that friends encouraged them and put pressure on them to try marijuana and other drugs. The social influence from the user and the supportive friendship provided an environment that encouraged others to abuse psychoactive substances. One of the participants said-

*“My friend circle motivated me a lot by giving examples like*, *look nothing happened to us*. *As they were taking the weed*, *nitrogen tablets*, *brown sugar for a long time*, *and life is a matter of enjoyment*, *and ultimately I got involved*.*”* [Male participant 23 years, Chhetri, FGD].

Some participants of FGDs and IDI said that friend circles played a key role in alcohol initiation, by highlighting the relationship with alcohol and culture. Most participants from ethnic groups mentioned that they started with homemade alcohol and later their curiosity to try other forms of psychoactive substances (weed, brown sugar) increased. One participant said-

*“I got involved in drugs (weed) via my friend circle which was created at school*. *I started at the age of 12*. *They pressured me showing ethnic relations to alcohol -Khana kei hudaina…jaat le deko yar [*Have it, it’s the gift given by your ethnicity, English translation*] and I felt it true and started taking it*.*”* [Male participant, 24 years, indigenous, FGD].

The majority of the participants from IDIs mentioned that their friends encouraged them to use psychoactive substances to get relief from stress.

*“I asked for solutions with my friend*, *and they told me to take these drugs and will get relief from the tension*. *But*, *after getting into this (substance use)*, *it’s hard to withdraw*.*”* [Male participant, 23 years, Chhetri, FGD].

#### ii) Source of stress relief

Youth used psychoactive substances as a source of relief in order to alleviate frustration and stress.

Most of the participants of FGDs expressed that they used psychoactive substances as a coping strategy to deal with stress caused because of the break-up in a relationship and family problems:

*“I start using drugs to get relief from tension caused by study and financial hardship*.*”* [Male participant, 22 years, IDI].

A few participants said that they have seen people relaxed, and free of tension or negative feelings through the use of psychoactive substances, which motivated them to try substances:

*“Psychoactive substance use gave me energy and a sense of belief that stress*, *and problems can be solved”*. [Male Participant, 19 years, Brahmin, FGD].

#### iii) Financial challenges

Most of the participants cited financial challenges as a pertinent factor behind psychoactive substance use. One participant expressed:

*“We* [my family] *took a loan from someone in our community with an interest of 10% per month to build a home*. *We paid interest and huge amount but at last*, *he* [loan giver] *did not return our property*. *This put me in tension and to cope with that I started taking drugs*.*”* [Male Participant, 23 years, IDI].

Additionally, few participants described the financial challenges faced by Nepalese students in foreign countries increases the risk of drug abuse. One participant of IDI shared the following experience:

*“I was in Australia and was not able to find a job and family could not support me financially as they already had loans and I could not cope up with the situations which forced me to involve in using drugs*.*”* [Male participant, 22 years, IDI].

### C. Physical environment

The physical environment played a substantial role in shaping the attitudes and behaviors of individuals and influencing norms and values. In this context, two key themes emerged relating to the physical environment: the accessibility and availability of psychoactive substances in the surrounding environment, and the lack of monitoring and enforcements by authorities.

#### i) Accessibility and availability of psychoactive substances in the surrounding environment

Participants identified the easy availability of psychoactive substances in the home, neighborhood, local markets, or near city areas where psychoactive substances like alcohol, cigarettes, marijuana and illegal drugs are available to youth. Participants said that this type of environment encourage youth to try psychoactive substances at first, which leads to sustained psychoactive substance use. Participants of FGDs mentioned that tobacco, alcohol, and substances like nitrogen tablets, brown sugar and marijuana were readily available in the local pharmacy and grocery shops (which is illegal in Nepal) and can be purchased very easily using peer network. One of the participants said:

*“I started taking Nitrogen tablets in beginning*, *later on*, *brown sugar*, *opium*. *It is easily available in the Butwal markets*.*”* [Male participant, 19 years, Indigenous, FGD].

Similarly, one participant from IDI mentioned his experience of buying psychoactive substances even from pharmacy shops.

*“Drugs are easily available from Butwal pharmacy*, *they* [pharmacists] *do everything for money*.*”* [Male participant, 21 years, IDI].

Participants from IDI and FGDs also stated that the accessibility of psychoactive substances at the nearby Indo-Nepal border facilitated adolescent initiation of psychoactive substances (such as Nitrogen tablets, brown sugar, cocaine, heroin). Over-the-counter medicine consumption is common in both rural and urban setting of Nepal because people can get medicines directly from unregulated non-pharmacist run registered/unregistered ‘drug shops’ similar to grocery shops. Some participants of IDIs and FGDs specifically referred to the availability and use of cough syrups (Phensedyl), Dendrites (adhesive synthetic product for footwear), fevicol glue (a synthetic adhesive used for woodworks) and sedatives like Nembutal, zolpidem and Seconal.

#### ii) Monitoring and enforcement

Most of the participants in this study (in both FGDs and IDIs) believed that political bodies, local policemen, shopkeepers (particularly grocery stores), pharmacists and border personnel at the Indo-Nepal border enable environments that allow for the availability of psychoactive substances (including nitrogen tablets, brown sugar and heroin). Additionally, participants noted the role that authorities play in the drug smuggling process. One participant of IDI shared his experience who had seen some police officials involved in trading psychoactive substances and drug paraphernalia:

*“Few policemen are also drug addicts*, *and they know the supply mechanism*. *Under* [policemen] *support drugs are available in the market*. *Besides policemen*, *there are many high-profile people in drug trading in Butwal*.*”* [Male Participant, 23 years, IDI].

A few of the participants from FGD mentioned that it was easy to escape from the police by giving bribes if they were caught in drug trafficking:

*“I was caught once by the police but bribing police with was enough to escape from border*.*”* [Male participant, 26 years, FGD].

Most participants in IDI described that the inadequate monitoring of psychoactive substances in society such as public bars, pubs, restaurants, and sometimes brothels, where psychoactive substances were sold and used on site. Some of the participants of IDI also mentioned that adolescents and women from marginalized communities (Indigenous and Dalit community) were generally involved in the cross-border trafficking of drugs, as officials overlooked traffickers either because of their age or physical status (i.e., those who appeared poor and vulnerable were allowed to travel without checking).

*“I used to wrap the drugs in the plastic and keep it inside the shoe insole so that police can’t find it*. *Using this technique*, *I transported drugs to Butwal*.*”* [Male participant, 22 years, Indigenous, IDI].

A small number of participants described taking risks to hide or transport drugs to be undetected. This included getting involved in trafficking psychoactive substances through swallowing small plastic pouches or mixing psychoactive substances with other large food items:

*“I got into cross-border drug supply since the age of 7 years old*. *They* [smugglers] *trained me in swallowing brown sugar sachets in India*. *After crossing border (India-Nepal border) I excrete those sachets in Nepal through stool*. *This was the safest way to escape the border police*.*”* [Male participant, 11 years, Dalit Community, FGD].

### D. Academic environment

The school environment also played a vital role in students’ lives. Youths spent a large amount of time in school among other fellow students and teachers. Participants from FGDs and IDI explicitly mentioned that failure to achieve good grades and lack of support from teachers was the primary reason to leave school and use psychoactive substances.

*“Actually*, *in my case*, *the schoolteacher never supported me and used to give my example in the class*. *This guy* [indicating participant*] is good for nothing*, *and he cannot do anything in the future*. *With all this*, *I opt out of school and later got involved in using drugs*.*”* [Male participant, 19 years, Indigenous, FGD].

*“I was weak in study and my school friends suggested that using diazepam and morphine [pharmaceutical drugs] can improve concentration on the study*. *I used these drugs for sometimes and later it became too hard to withdraw [*stop drug use*]*.*”* [Male participant, 16 years, Chhetri, FGD].

### E. Influence of media

Participants reported that digital media and television programs glamorized psychoactive substance use, which directly influenced especially, adolescents and adults to try psychoactive substances. Some participants in the FGDs mentioned the use of psychoactive substances by celebrities in movies or on social media pages, while some participants from IDIs said that their use of psychoactive substances (tobacco, alcohol, or marijuana) was to feel like an actor in a movie or television-serial. This copying practice was perceived as fun or exciting and led to the experimentation of psychoactive substances. In some cases, this led to sustained use of substances and abuse of psychoactive substances. One of the participants of FGD said, he used to have various non-prescribed medicines and available materials like dendrites (glue) to have fun and a good feeling, which turned him to be psychoactive substances abuser:

“*I used dendrites*, mixed different medical drugs*. *I used to try different pharmaceutical drugs to get in the mood*.*”* [Male Participants, 30 years, Indigenous, FGD].

*Note. Mixing of the liquid drugs used to treat kidney disease patients to get ‘a good kick’.

## Discussion

This study identified crucial socio-cultural, individual, physical environment, academic environments and the influence of media, as key fueling factors of psychoactive substance use among Nepalese youths of this study. Despite a small study group, consisting of 20 participants, this study presents a rich source of information on the factors that influence youth initiation and use of psychoactive substances in Nepal. This research reports on the experiences of youth who had been at rehabilitation centers (and some from a very young age). While this may be unique to the current sample, it will be important that future research explores whether these themes emerge in a sample of youth from the general population of Nepal. This research serves as an important starting point to better understand these behaviors and develop appropriate interventions to reduce the harm caused by use of psychoactive substances.

The findings of this study identified the family, culture, and societal environment, where the growth and development of individuals take place, as an initiating factor for psychoactive substance use among youth in Nepal. Parental separation, detachment from family members and poor parental supervision contribute to stress among adolescents, which encouraged them to use psychoactive substances. This finding is consistent with other research that found family environment as a determining factor for the engagement of youths in psychoactive substance handling and use [24, 25]. Studies have also reported high use of psychoactive substances among adolescents who return from rehabilitation centres (due to peer pressure, family level conflict or adjustment within family, low self-confidence, and low acceptance by the society) [26, 27]. However, given the cultural nuances identified in this cohort, future research should seek to better understand how these societal factors interact with each other, as well as other factors that shapes pathways to psychoactive substance use among Nepalese youths.

This study also found ethnicity as a key driver for psychoactive substance use among Nepalese youth. Prior research from India have highlighted that alcohol use among some indigenous communities is strongly connected to cultural rituals where part of alcohol is offered to the god and the remaining is consumed by the devotees as prasad (a devotional offering made to a god and later shared by devotee) [28, 29]. The cultural acceptances of psychoactive substance use in some ethnic groups have previously documented in studies from Nepal [17, 30]. However, previous studies from Nepal reported that the non-indigenous communities were more involved in psychoactive substance use compared to indigenous people [13, 31]. This might be because of the cultural differences where one specific community culture provides a favorable environment for youth of other communities to use psychoactive substances such as alcohol, while these activities are less accepted in their own community.

It was perhaps no surprise that peer groups appeared to play a very influential role for the initiation and continued use of psychoactive substances, especially among youths, given previous studies conducted in Nepal and other countries showed the influence of peers to use psychoactive substances [32–35]. Since adolescents and youth share special bonding rituals with friends, it is easier to be influenced by the behaviors of friends and difficult to reject offers of psychoactive substance use [36]. In addition, the influence to use psychoactive substance from their peer circle can be explained by societal cognitive theory which highlights some of the factors like environment and others action play a vital role in behavioral change [37]. Also, studies have reported factors such as appreciation shown by peer circle can induce the psychoactive substance use [38].

This study also observed the use of psychoactive substance in relation to stress management. This supports other evidence that shows stressful events and traumatic exposure creates neurological shifting which may decrease behavioral control and accelerates the risk of maladaptive behavior [39]. This is also supported by other studies that found use of psychoactive substance as a source of relaxation and relief among students [36, 40]. The financial status of the individual and family was found to be contributing factor to increased use of psychoactive substances. Low financial status and economic instability generates stress and acts as a stimulant of psychoactive substance initiation. A previous study reported interconnection between economic crisis and increased prevalence of psychoactive substance abuse, needle and syringe sharing [41, 42]. Beside these, the misuse of having higher financial capability was also associated with the high connection with psychoactive substance user gangs and increased psychoactive substance use like alcohol, marijuana which is consistent with other studies [43–45].

Easy availability of psychoactive substances in local markets was identified as an enabling factor for use among youths. Readily available alcohol and tobacco in the household was the first choice of psychoactive substance among our study participants which was also reported in previous studies [17, 46]. Misuse of pharmacological drugs was reported by many participants in the present study. Similarly, misuse of psychotropic drugs and the facilitation of over-the-counter drugs were found in other studies [47]. Furthermore, minor opiates and pain medicines (Temazepam, Flurazepam) were found to be commonly misused by healthcare students for getting kick [32]. In addition, poor monitoring of security personnel in the Nepal-India border aids to cross-border trafficking and easy availability of psychoactive substance in the local market [48]. Poor academic performance and grades cause stress among youth which increases risk of psychoactive substance use as a coping mechanism. Such students engage in psychoactive substance use with hope of gaining better scores and to get relief from the dissatisfaction caused by the poor academic achievements [36, 49, 50]. The present study showed academic pressure as an enabler for psychoactive substance abuse among youth and this highlights the importance of school/college-based intervention to improve study environment. This can be done by creating school policies to recognize students with high level of stress, inclusion of extra curriculum chapters such as stress management education, encouragement, good communication and motivation to the needy ones [51]. In addition, to minimize the psychological distress among students, incorporation of life and social skills training and problem-based learning skills in an academic curriculum which may help students to function independently and choose right decisions [52]. According to United Nations Office on Drugs and Crime, skills-based health education includes the interactive sessions by skilled teacher which helps in building personal and social abilities to deal with daily life circumstances that one faces [53].

The present study revealed the influence of media and celebrities in promoting psychoactive substance use among youth. Media often portrays psychoactive substances as a source of entertainment through role play which indirectly fosters psychoactive substance use [54]. It is highly likely that psychoactive substances use among youths increases when media artists publicly demonstrate drinking alcohol, smoking cigarette or using drugs to show aggressive behavior, cope with personal circumstances such as relationship break-up, financial challenges or demonstrate the power in their networks. Prior studies have demonstrated the role of media in promoting substance use among youth [55, 56].

Youth who are involved in psychoactive substance use were found to be indulged with various unlawful behaviors such as involvement in vigorous and gang sex, criminal mischief and in burglary to collect money to have psychoactive substances [57–60].

Our findings suggest the need for interventions that deliver cultural friendly health literacy initiatives to be operated at different levels such as school, community, youth clubs to educate youths on the detrimental effects of using psychoactive substances and providing information on the available services to help them to get rid of psychoactive substance use. In addition, youth identified as at-risk should be supported through targeted activities that seek to identify and prevent factors that enable pathways to psychoactive substance use. We also suggest the need for strong implementation of policies that prohibit the psychoactive substances being sold to children below 18 years. Further, we suggest the need for national level research focusing on youth to understand the social and structural determinants of psychoactive substance use among Nepalese youths. The findings from such study may help the government of Nepal to develop policies and strategies to curb psychoactive substance use among the youths.

### Strengths and limitations

Strengths of this study include that this is one of the first qualitative studies conducted in Nepal that seeks to understand the factors that influence psychoactive substance use initiation, rather than collecting survey data. This study also provides starting point for additional in-depth qualitative studies based on findings AND knowledge developed in this study. One of the key limitations of this study was a small study conducted within participants of one district from participants who were in rehabilitation centers, therefore the findings may not be generalizable to the broader population of the country. Another limitation is that we did not collect data from family and community members, teachers and other stakeholders who could have provided more insights on this research area.

## Conclusion

This study explored multiple factors that influenced initiation and engagement in psychoactive substance use among young people in the Rupandehi district of Nepal. The findings from this study may assist policymakers to design multi-sectoral responses to prevent harm from psychoactive substance related issues among Nepalese youth. These preliminary findings suggest there is a need for additional research with multilevel stakeholders, such as youths, family members, schools and community based local organizations and police officials to better understand psychoactive substance consumption behaviors. Future work should seek to identify interventions that address these factors to reduce youth initiation and using psychoactive substances and preventing subsequent harms.
